# [Retracted] Tumor suppressor miR‑613 induces cisplatin sensitivity in non‑small cell lung cancer cells by targeting GJA1

**DOI:** 10.3892/mmr.2024.13291

**Published:** 2024-07-15

**Authors:** Jianhua Luo, Yan Jin, Mengyuan Li, Liyang Dong

Mol Med Rep 23: 385, 2021; DOI: 10.3892/mmr.2021.12024

Following the publication of this paper, it was drawn to the Editor's attention by a concerned reader that certain of the colony formation assay data shown in Fig. 2F, the tumor images in Fig. 3A, the “NC” experiment for the Ki67 immunohistochemical staining experiment shown in Fig. 3E and the migration assay data in Fig. 4D were strikingly similar to data appearing in different form in other papers by different authors at different research institutes that had either already been published, or were under consideration for publication at around the same time.

Owing to the fact that the contentious data in the above article had already been published prior to its submission to *Molecular Medicine Reports*, the Editor has decided that this paper should be retracted from the Journal. The authors were asked for an explanation to account for these concerns, but the Editorial Office did not receive a reply. The Editor apologizes to the readership for any inconvenience caused.

